# Supplementary material for: Land-Use Type Drives Soil Population Structures of the Entomopathogenic Fungal Genus Metarhizium
Source: Microorganisms. 2021 Jun 25;9(7):1380. doi: 10.3390/microorganisms9071380 (PMC8303860; doi:10.3390/microorganisms9071380)
Supplement: Supplementary file 1 [file microorganisms-09-01380-s001.zip › Supplementary_Figures.pdf]

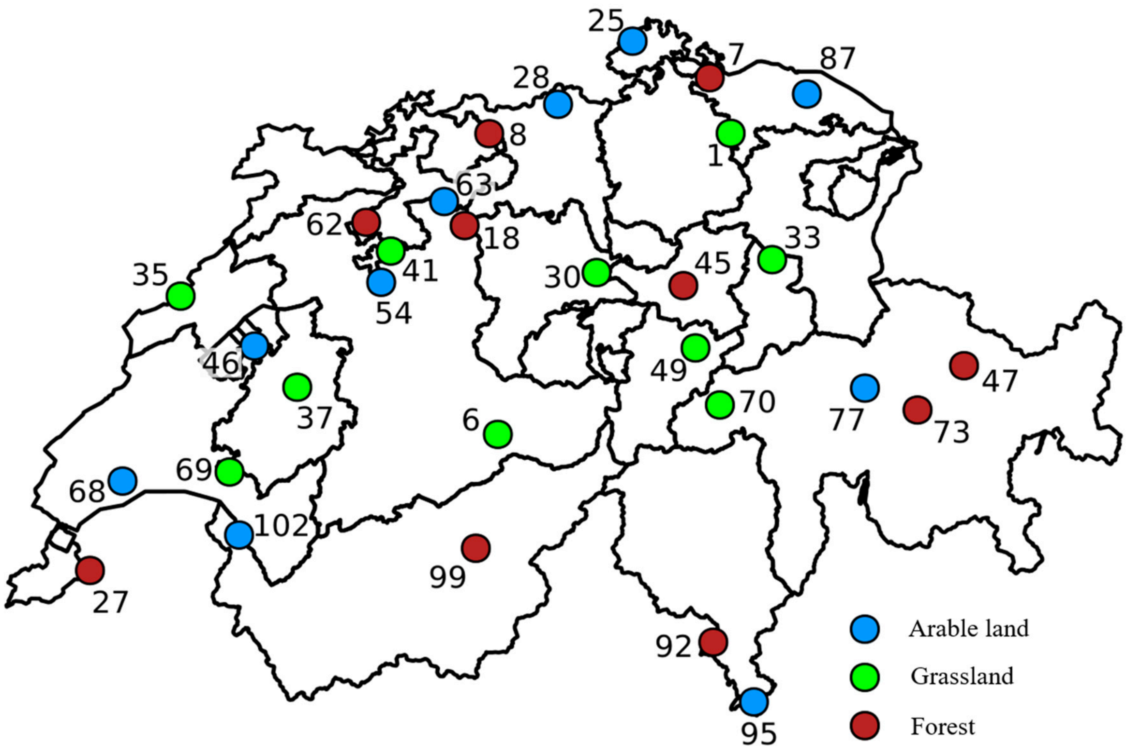

Figure S1. Georeferenced sampling sites of the three land-use types, comprising 10 arable land sites, 10 grassland sites and 10 forest sites (Gschwend et al., 2021, in press [46]).
