# Supplementary material for: Land-Use Type Drives Soil Population Structures of the Entomopathogenic Fungal Genus Metarhizium
Source: Microorganisms. 2021 Jun 25;9(7):1380. doi: 10.3390/microorganisms9071380 (PMC8303860; doi:10.3390/microorganisms9071380)
Supplement: Supplementary file 1 [file microorganisms-09-01380-s001.zip › Supplementary_Tables.pdf]

Table S1. Summary information of samples. (see Excel file)

Table S2. Site metadata information: description of environmental factors of each site. (see Excel file)

Table S3. Means and ANOVA of *Metarhizium* spp. colony forming units (CFU) g^{-1} of soil dry weight in each site. Affiliation of each site to one of the three abundance groups based on mean CFU is indicated.

Site	Abundance group[1)]	Land-use type	Mean±SD [2)]	Significance [3)]
**1**	Medium	Grassland	658.33±72.16	EF
**6**	Medium	Grassland	908.33±702.82	EF
**7**	Medium	Forest	1466.70±763.35	DEF
**8**	Medium	Forest	475.00±565.69	EF
**18**	Medium	Forest	2750.00±777.42	BCDEF
**25**	Low	Arable land	0.00±0.00	F
**27**	Low	Forest	0.00±0.00	F
**28**	Medium	Arable land	2208.30±791.49	CDEF
**30**	Medium	Grassland	566.67±225.46	EF
**33**	High	Grassland	4825.00±463.01	ABC
**35**	High	Grassland	5783.3±1703.2	AB
**37**	Medium	Grassland	2766.70±734.14	BCDEF
**41**	High	Grassland	4150.00±217.94	ABCD
**45**	Medium	Forest	1791.70±2433.70	CDEF
**46**	Low	Arable land	33.33±57.73	EF
**47**	Low	Forest	0.00±0.00	F
**49**	Medium	Grassland	3041.70±777.15	ABCDEF
**54**	Medium	Arable land	1375.00±626.50	DEF
**62**	Low	Forest	0.00±0.00	F
**63**	Low	Arable land	0.00±0.00	F
**68**	Low	Arable land	25.00±0.00	EF
**69**	High	Grassland	6362.50±1467.20	A
**70**	High	Grassland	5950.00±3356.10	A
**73**	Low	Forest	0.00±0.00	F
**77**	Medium	Arable land	1241.70±667.24	DEF
**87**	Medium	Arable land	1450.00±1244.70	DEF
**92**	Low	Forest	0.00±0.00	F
**95**	Medium	Arable land	3158.30±357.36	ABCDE
**99**	Low	Forest	0.00±0.00	F
**102**	Low	Arable land	100.00±50.00	EF

[1)] Low represents sites with <150 CFU g^{-1} of soil dry weight, medium between 150 and 4000 CFUs; high > 4000 CFUs. [2)] Mean values ± standard deviation. [3)] Different letters indicated significantly different abundance ($P<0.05$, Tukey test).

Table S4. Summary of 15 soil and environmental factors affecting the abundance groups observed among the 30 sites: high, medium, and low *Metarhizium* abundance (CFU g^{-1} of soil dry weight).

	**High**				**Medium**				**Low**				**ANOVA**		
	**Mean**	**SD**	**Minimum**	**Maximum**	**Mean**	**SD**	**Minimum**	**Maximum**	**Mean**	**SD**	**Minimum**	**Maximum**	**F-value**	***p*-value**	**Pattern[1)]**
Altitude (masl)	782.2	302.4	431.0	1105.0	760.7	395.4	336.0	1915.0	813.0	451.2	379.0	1655.0	0.2	0.8568	H=M=L
Basal respiration[2)]	1.3	0.2	0.9	1.7	1.4	1.1	0.5	5.3	1.6	1.1	0.5	4.6	0.5	0.6387	H=M=L
Clay (%)	24.8	7.0	12.5	33.0	24.6	8.8	11.5	42.0	24.5	15.9	5.8	59.0	0.0	0.9971	H=M=L
Silt (%)	42.3	8.4	34.3	55.0	37.9	8.2	25.0	51.5	39.6	13.6	18.6	59.8	1.0	0.3709	H=M=L
Sand (%)	33.0	14.2	12.0	50.8	37.6	10.0	18.0	54.0	35.9	21.2	11.0	71.0	0.5	0.6152	H=M=L
Soil skeleton (%)	3.5	4.7	0.0	11.3	2.8	1.8	0.0	5.1	3.0	3.7	0.0	11.2	0.2	0.796	H=M=L
pH ($CaCl_2$)	5.3	0.4	4.7	5.9	5.7	1.2	3.4	7.1	5.5	1.4	3.4	7.5	0.4	0.6665	H=M=L
Total Carbon ((%)	3.8	0.7	2.9	5.1	4.2	3.3	1.1	16.0	5.4	4.4	1.8	17.3	1.5	0.2216	H=M=L
Organic Carbon (%)	3.8	0.7	2.9	5.1	4.2	3.3	1.1	16.0	5.2	4.5	1.4	17.3	1.1	0.3487	H=M=L
Total Nitrogen (%)	0.4	0.1	0.3	0.6	0.4	0.2	0.1	0.9	0.3	0.2	0.1	1.1	0.3	0.7226	H=M=L
C:N_ratio	9.6	0.4	8.9	10.0	11.1	3.1	8.0	17.7	14.2	5.5	8.6	27.0	8.7	**0.0004**	**H=M≠L**
Bulk density (kg/dm^3)	1.0	0.1	0.9	1.1	1.0	0.3	0.2	1.4	0.9	0.3	0.3	1.3	0.9	0.4168	H=M=L
DNA (mg/kg)	47.2	15.0	27.0	74.0	41.6	20.7	14.0	95.0	37.1	26.8	13.0	117.0	1.1	0.3408	H=M=L
MAT[3)] (ºC)	6.5	3.4	1.7	11.0	7.2	3.9	-2.3	11.0	7.9	3.4	0.2	12.3	0.8	0.4653	H=M=L
MAP[4)] (mm)	1496.2	321.0	1090.0	1910.0	1342.4	363.2	962.0	2140.0	1116.7	368.5	528.0	1838.0	6.8	**0.0019**	**H=M≠L**

[1)]Significant differences of pairwise test between the proportion of CFUs ($P<0.05$, Tukey test). [2)]Basal Respiration: mg CO_2-C Jg^{-1} TS h^{-1}. [3)]MAT: mean annual temperature; [4)]MAP: mean annual precipitation.

Table S5. Summary of 15 soil and environmental factors affecting on two *Metarhizium* abundance (CFU g^{-1} of soil dry weight) groups, medium and low, **in arable land**

	**Medium**				**Low**				**ANOVA**		
	**Mean**	**SD**	**Minimum**	**Maximum**	**Mean**	**SD**	**Minimum**	**Maximum**	**F-value**	***p*-value**	**Pattern[1)]**
Altitude (masl)	549.4	167.9	336	830	449.6	55.571	379	545	4.78	**0.0374**	**M≠L**
Basal respiration[2)]	0.7467	0.2326	0.45	1.06	0.7867	0.2482	0.47	1.09	0.21	0.6523	M=L
Clay (%)	17.55	4.5808	11.5	23.75	32.53	19.135	5.75	59	8.69	**0.0064**	**M≠L**
Silt (%)	40.25	6.6882	34	51.25	45.11	9.7516	30	59.75	2.53	0.1227	M=L
Sand (%)	42.2	9.8299	31	54	22.36	11.363	11	36.05	26.15	**0**	**M≠L**
Soil skeleton (%)	3.98	0.7618	3.1	4.9	0.72	0.7702	0	2	135.85	**0**	**M≠L**
pH ($CaCl_2$)	6.5667	0.488	5.7	7.1	6.7333	0.8748	5.7	7.5	0.42	0.5245	M=L
Total Carbon ((%)	1.958	0.6743	1.14	3.17	2.792	0.8752	1.76	4.29	8.55	**0.0068**	**M≠L**
Organic Carbon (%)	1.954	0.6748	1.14	3.17	2.302	0.6289	1.43	3.03	2.13	0.1551	M=L
Total Nitrogen (%)	0.218	0.094	0.12	0.39	0.2527	0.084	0.13	0.35	1.13	0.296	M=L
C:N_ratio	9.202	0.6412	8.03	10	9.3233	0.7301	8.58	10.98	0.23	0.6324	M=L
Bulk density (kg/dm^3)	1.168	0.1709	0.94	1.38	1.156	0.1291	0.95	1.32	0.05	0.8298	M=L
DNA (mg/kg)	25.467	9.0543	14	45	17.933	3.7696	13	26	8.85	**0.006**	**M≠L**
MAT[3)] (ºC)	9.24	1.0927	7.9	11	8.48	2.4699	3.9	10.9	1.19	0.2851	M=L
MAP[4)] (mm)	1134.6	209.7	986	1532	1173.8	352.97	905	1838	0.14	0.7143	M=L

[1)]Significant differences of pairwise test between the proportion of CFUs ($P<0.05$, Tukey test). [2)]Basal Respiration: mg CO_2-C Jg^{-1} TS h^{-1}; [3)]MAT: mean annual temperature; [4)]MAP: mean annual precipitation.

Table S6. : Summary of 15 soil and environmental factors affecting two *Metarhizium* abundance (CFU g^{-1} of soil dry weight) groups, high and medium, in **grassland**.

	**High**				**Medium**				**ANOVA**		
	**Mean**	**SD**	**Minimum**	**Maximum**	**Mean**	**SD**	**Minimum**	**Maximum**	**F-value**	***p*-value**	**Pattern[1)]**
Altitude (masl)	782.2	302.37	431	1105	984.4	520.4	537	1915	1.69	0.2038	H=M
Basal respiration[2)]	1.2867	0.2094	0.94	1.68	1.2133	0.2462	0.86	1.59	0.77	0.387	H=M
Clay (%)	24.75	7.0432	12.5	33	26.3	6.8101	19.25	35	0.38	0.545	H=M
Silt (%)	42.3	8.4003	34.25	55	35.575	7.9033	27	49.875	5.1	**0.0319**	**H≠M**
Sand (%)	32.95	14.22	12	50.75	38.125	8.8037	25.625	47.75	1.44	0.2408	H=M
Soil skeleton (%)	3.48	4.6749	0	11.3	2.14	1.3773	0	4.1	1.13	0.296	H=M
pH ($CaCl_2$)	5.34	0.368	4.7	5.9	5.02	0.758	3.9	6.2	2.16	0.1525	H=M
Total Carbon ((%)	3.782	0.6824	2.89	5.1	4.1907	1.1506	2.88	5.7	1.4	0.2467	H=M
Organic Carbon (%)	3.782	0.6824	2.89	5.1	4.1907	1.1506	2.88	5.7	1.4	0.2467	H=M
Total Nitrogen (%)	0.396	0.0871	0.29	0.56	0.438	0.1132	0.31	0.62	1.3	0.2644	H=M
C:N_ratio	9.6207	0.3855	8.91	10.03	9.584	1.2292	8.32	11.89	0.01	0.913	H=M
Bulk density (kg/dm^3)	0.992	0.0646	0.9	1.08	0.988	0.1601	0.72	1.13	0.01	0.9292	H=M
DNA (mg/kg)	47.2	14.977	27	74	49.533	18.527	15	85	0.14	0.7073	H=M
MAT[3)] (ºC)	6.52	3.4439	1.7	11	4.36	5.2367	−2.3	9.8	1.78	0.1927	H=M
MAP[4)] (mm)	1496.2	321	1090	1910	1524.6	290.24	1229	1979	0.06	0.8012	H=M

[1)]Significant differences of pairwise test between the proportion of CFUs ($P<0.05$, Tukey test). [2)]Basal Respiration: mg CO_2-C Jg^{-1} TS h^{-1}; [3)]MAT: mean annual temperature; [4)]MAP: mean annual precipitation.

Table S7. Summary of 15 soil and environmental factors affecting two *Metarhizium* abundance (CFU g^{-1} of soil dry weight) groups, medium and low, in **forest.**

	**Medium**				**Low**				**ANOVA**		
	**Mean**	**SD**	**Minimum**	**Maximum**	**Mean**	**SD**	**Minimum**	**Maximum**	**F-value**	***p*-value**	**Pattern[1)]**
Altitude (masl)	745.25	269.86	525	1180	1115.8	408.73	505	1655	7.6	**0.0101**	**M≠L**
Basal respiration[2)]	2.5308	1.5974	0.78	5.29	2.2294	1.073	0.86	4.64	0.38	0.5403	M=L
Clay (%)	31.125	9.1437	18.75	42	17.758	8.27	7	30.5	17.3	**0.0003**	**M≠L**
Silt (%)	37.813	9.9864	25	51.5	35.013	14.775	18.575	52	0.33	0.571	M=L
Sand (%)	31.063	8.6885	18	39	47.229	20.96	17.5	71	6.35	**0.0177**	**M≠L**
Soil skeleton (%)	2.275	2.4106	0	5.1	4.9333	4.09	0	11.2	4.09	0.0528	M=L
pH ($CaCl_2$)	5.3	1.5196	3.4	6.8	4.4944	0.8475	3.4	5.6	3.48	0.0727	M=L
Total Carbon ((%)	7.0942	4.7001	3.01	15.95	7.5956	4.9793	2.61	17.31	0.08	0.7844	M=L
Organic Carbon (%)	7.0292	4.7387	2.88	15.95	7.5956	4.9793	2.61	17.31	0.1	0.7581	M=L
Total Nitrogen (%)	0.4375	0.251	0.24	0.9	0.4228	0.3089	0.19	1.11	0.02	0.8917	M=L
C:N_ratio	15.457	2.2184	12.01	17.72	18.232	4.2808	13.65	26.97	4.25	**0.0488**	**M≠L**
Bulk density (kg/dm^3)	0.7075	0.3253	0.21	1.07	0.6783	0.3073	0.26	1.07	0.06	0.8053	M=L
DNA (mg/kg)	51.833	22.514	27	95	53.056	27.243	21	117	0.02	0.8985	M=L
MAT[3)] (ºC)	8.25	1.1115	6.7	9.7	7.3833	4.0561	0.2	12.3	0.52	0.4784	M=L
MAP[4)] (mm)	1374.5	472.46	962	2140	1069.2	384.36	528	1796	3.78	0.0618	M=L

[1)]Significant differences of pairwise test between the proportion of CFUs (P<0.05, Tukey test). [2)]Basal Respiration: mg CO_2-C Jg^{-1} TS h^{-1}; [3)]MAT: mean annual temperature; [4)]MAP: mean annual precipitation.

Table S8. Overall permutational multivariate analysis of variance (PERMANOVA) for individual soil and environmental factors, which significantly affect *M. brunneum* population structure among three land-use types. Analyses were based on Bray-Curtis distances.

Factor	Pseudo-*F* statistic	R^2	*p*-value
C:N ratio	6.416	0.252	0.0001
BR (Basal Respiration) (mg CO_2-C Jg^{-1} TS h^{-1})	2.980	0.135	0.0071
Organic Carbon (%)	2.601	0.120	0.0039
Total Carbon (%)	2.477	0.115	0.0055
Bulk density (kg/dm^3)	2.912	0.133	0.0056
Clay (%)	1.638	0.079	0.1317
DNA (mg/kg)	1.951	0.093	0.0761
Sand (%)	1.133	0.056	0.3298
Soil skeleton volume (%)	0.813	0.041	0.5821
Total Nitrogen (%)	1.418	0.069	0.1948
pH (CaCl2)	1.249	0.061	0.2738
Altitude (masl)	1.271	0.062	0.2632
Silt (%)	0.918	0.045	0.4873
MAT (mean annual temperature (ºC)	0.605	0.032	0.7211
MAP (mean annual precipitation) (mm)	0.568	0.029	0.7661
**Land_use type**	5.364	0.373	0.0002

Table S9. Overall permutational multivariate analysis of variance (PERMANOVA) for individual soil and environmental, factors which significantly affect *M. brunneum* population structure between arable land and grassland. Analyses were based on Bray-Curtis distances.

Factor	Pseudo-*F* statistic	R^2	*p*-value
C:N ratio	1.714	0.106	0.1149
BR (Basal Respiration) (mg CO_2-C Jg^{-1} TS h^{-1})	1.973	0.116	0.0928
Organic Carbon (%)	1.991	0.117	0.0861
Total Carbon (%)	1.561	0.094	0.1657
Bulk density (kg/dm^3)	1.375	0.083	0.2375
Clay (%)	1.724	0.103	0.1265
DNA (mg/kg)	2.062	0.120	0.0841
Sand (%)	1.229	0.075	0.2955
Soil skeleton volume (%)	0.962	0.060	0.4503
Total Nitrogen (%)	1.855	0.110	0.1073
pH (CaCl2)	1.483	0.089	0.1920
Altitude (masl)	1.654	0.099	0.1459
Silt (%)	1.449	0.088	0.2063
MAT (mean annual temperature (ºC)	0.698	0.044	0.6205
MAP (mean annual precipitation) (mm)	0.685	0.043	0.6385
**Land_use type**	2.538	0.144	0.0513

Table S10. Overall permutational multivariate analysis of variance (PERMANOVA) for individual soil and environmental, factors which significantly affect *M. brunneum* population structure within arable land. Analyses were based on Bray-Curtis distances.

Factor	Pseudo-*F* statistic	R^2	*p*-value
C:N ratio	0.150	0.029	0.9821
BR (Basal Respiration) (mg CO_2-C Jg^{-1} TS h^{-1})	1.312	0.207	0.3016
Organic Carbon (%)	0.884	0.150	0.5563
Total Carbon (%)	0.836	0.143	0.5891
Bulk density (kg/dm^3)	0.941	0.158	0.5190
Clay (%)	0.950	0.159	0.5240
DNA (mg/kg)	0.302	0.057	0.9350
Sand (%)	1.243	0.199	0.3258
Soil skeleton volume (%)	0.547	0.098	0.8032
Total Nitrogen (%)	0.666	0.117	0.6980
pH (CaCl2)	0.997	0.166	0.4829
Altitude (masl)	0.230	0.440	0.9433
Silt (%)	0.735	0.128	0.6758
MAT (mean annual temperature (ºC)	0.788	0.136	0.6056
MAP (mean annual precipitation) (mm)	0.468	0.085	0.8518

Table S11. Overall permutational multivariate analysis of variance (PERMANOVA) for individual soil and environmental, factors which significantly affect *M. brunneum* population structure within grassland. Analyses were based on Bray-Curtis distances.

Factor	Pseudo-*F* statistic	R^2	*p*-value
C:N ratio	5.264	0.263	0.0074
BR (Basal Respiration) (mg CO_2-C Jg^{-1} TS h^{-1})	0.534	0.062	0.7186
Organic Carbon (%)	0.619	0.071	0.6421
Total Carbon (%)	0.619	0.071	0.6488
Bulk density (kg/dm^3)	2.098	0.207	0.0893
Clay (%)	0.603	0.070	0.6732
DNA (mg/kg)	0.829	0.093	0.5163
Sand (%)	1.755	0.179	0.1545
Soil skeleton volume (%)	0.834	0.044	0.5633
Total Nitrogen (%)	0.332	0.039	0.8745
pH (CaCl2)	1.854	0.129	0.3475
Altitude (masl)	2.120	0.209	0.0859
Silt (%)	2.396	0.230	0.0711
MAT (mean annual temperature (ºC)	0.999	0.111	0.4152
MAP (mean annual precipitation) (mm)	0.394	0.046	0.8208

Table S12. Overall permutational multivariate analysis of variance (PERMANOVA) for individual soil and environmental, factors which significantly affect *M. brunneum* population structure within forest. Analyses were based on Bray-Curtis distances.

Factor	Pseudo-*F* statistic	R^2	*p*-value
C:N ratio	1.813	0.475	0.2083
BR (Basal Respiration) (mg CO_2-C Jg^{-1} TS h^{-1})	3.058	0.604	0.1667
Organic Carbon (%)	2.357	0.541	0.2083
Total Carbon (%)	2.361	0.541	0.2083
Bulk density (kg/dm^3)	3.301	0.622	0.2500
Clay (%)	1.158	0.366	0.4167
DNA (mg/kg)	3.962	0.664	0.1667
Sand (%)	1.077	0.350	0.5000
Soil skeleton volume (%)	0.333	0.142	0.8333
Total Nitrogen (%)	2.168	0.520	0.2917
pH (CaCl2)	0.239	0.107	1
Altitude (masl)	1.823	0.476	0.3330
Silt (%)	0.123	0.058	1
MAT (mean annual temperature (ºC)	3.854	0.658	0.1667
MAP (mean annual precipitation) (mm)	2.301	0.535	0.25

Table S13. Overall permutational multivariate analysis of variance (PERMANOVA) for individual soil and environmental factors which significantly affect *M. robertsii* population structure among three land-use types. Analyses were based on Bray-Curtis distances.

Factor	Pseudo-*F* statistic	R^2	*p*-value
C:N ratio	0.749	0.076	0.5366
BR (Basal Respiration) (mg CO_2-C Jg^{-1} TS h^{-1})	1.915	0.175	0.1327
Organic Carbon (%)	1.360	0.131	0.2562
Total Carbon (%)	1.892	0.173	0.1403
Bulk density (kg/dm^3)	1.934	0.176	0.1333
Clay (%)	2.022	0.183	0.1249
DNA (mg/kg)	0.669	0.069	0.5883
Sand (%)	2.228	0.198	0.0930
Soil skeleton volume (%)	4.433	0.330	0.0098
Total Nitrogen (%)	1.311	0.127	0.2576
pH (CaCl2)	0.443	0.046	0.7663
Altitude (masl)	2.181	0.195	0.0838
Silt (%)	0.651	0.067	0.5977
MAT (mean annual temperature (ºC)	0.605	0.063	0.6520
MAP (mean annual precipitation) (mm)	0.910	0.918	0.4504
**Land-use type**	0.986	0.098	0.4665

Table S14. Overall permutational multivariate analysis of variance (PERMANOVA) for individual soil and environmental factors, which significantly affect *M. robertsii* population structure within arable land. Analyses were based on Bray-Curtis distances.

Factor	Pseudo-*F* statistic	R^2	*p*-value
C:N ratio	0.964	0.161	0.4599
BR (Basal Respiration) (mg CO_2-C Jg^{-1} TS h^{-1})	0.978	0.163	0.4595
Organic Carbon (%)	0.667	0.117	0.6476
Total Carbon (%)	1.458	0.225	0.3230
Bulk density (kg/dm^3)	0.753	0.130	0.6103
Clay (%)	2.107	0.296	0.1099
DNA (mg/kg)	0.799	0.137	0.5464
Sand (%)	1.862	0.271	0.1405
Soil skeleton volume (%)	1.199	0.193	0.3008
Total Nitrogen (%)	0.655	0.115	0.6175
pH (CaCl2)	0.902	0.152	0.4794
Altitude (masl)	1.482	0.228	0.2651
Silt (%)	0.635	0.112	0.6734
MAT (mean annual temperature (ºC)	0.454	0.083	0.8425
MAP (mean annual precipitation) (mm)	1.738	0.258	0.2242

Table S15. Overall permutational multivariate analysis of variance (PERMANOVA) for individual soil and environmental, factors which significantly affect *M. robertsii* population structure within grassland. Analyses were based on Bray-Curtis distances.

Factor	Pseudo-*F* statistic	R^2	*p*-value
C:N ratio	0.307	0.133	0.8333
BR (Basal Respiration) (mg CO_2-C Jg^{-1} TS h^{-1})	0.267	0.118	0.6667
Organic Carbon (%)	0.077	0.037	0.8333
Total Carbon (%)	0.077	0.037	0.8333
Bulk density (kg/dm^3)	2.190	0.522	0.1667
Clay (%)	0.158	0.073	0.5
DNA (mg/kg)	0.514	0.204	0.5
Sand (%)	0.302	0.131	0.8333
Soil skeleton volume (%)	5.323	0.726	0.3333
Total Nitrogen (%)	0.074	0.036	0.8333
pH (CaCl2)	1.112	0.357	0.5
Altitude (masl)	2.413	0.546	0.3333
Silt (%)	0.856	0.299	0.5
MAT (mean annual temperature (ºC)	1.007	0.334	0.5
MAP (mean annual precipitation) (mm)	0.265	0.117	0.8333

Table S16: Summary of environmental factors at the ten sites of each land-use type.

	Arable land			Grassland			Forest			ANOVA[1)]		
	mean	min	max	mean	min	max	mean	min	max	F	*p*-value	Pattern[2)]
**Site characteristics**												
Altitude [masl]	499.5	336.0	830.0	883.3	431.0	1915.0	967.6	505.0	1655.0	4.8	0.0165	A<G=F
Clay [%]	25.0	5.8	59.0	25.5	12.5	35.0	23.1	7.0	42.0	0.1	0.8943	A=G=F
Silt [%]	42.7	30.0	59.8	38.9	27.0	55.0	36.1	18.6	52.0	1.0	0.3993	A=G=F
Sand [%]	32.3	11.0	54.0	35.5	12.0	50.8	40.8	17.5	71.0	0.7	0.4927	A=G=F
Soil skeleton [%]	2.4	0.0	4.9	2.8	0.0	11.3	3.9	0.0	11.2	0.6	0.5640	A=G=F
MAT[3)] [°C]	8.9	3.9	11.0	5.4	-2.3	11.0	7.7	0.2	12.3	2.5	0.1023	A=G=F
MAP[4)] [mm]	1154.2	905.0	1838.0	1510.4	1090.0	1979.0	1191.3	528.0	2140.0	2.9	0.0711	A=F=G
**Yearly measurements[5)]**												
pH	6.6	5.6	7.5	5.2	3.8	6.3	4.8	3.3	6.9	10.6	0.0004	A>G=F
C_{tot} [%]	2.4	1.1	4.5	4.1	2.6	7.0	7.3	2.4	18.3	7.7	0.0023	A=G<F
C_{org} [%]	2.2	1.1	3.4	4.1	2.6	7.0	7.3	2.4	18.3	8.3	0.0015	A=G<F
N_{tot} [%]	0.2	0.1	0.5	0.4	0.3	0.7	0.4	0.2	1.1	3.2	0.0549	A=G=F
C/N	9.0	6.7	11.2	9.6	7.4	12.5	17.8	11.3	27.5	40.0	< 0.0001	A=G<F
Bulk density [kg/dm^3]	1.2	0.6	1.5	1.0	0.7	1.2	0.7	0.2	1.2	14.9	< 0.0001	A>G>F
Basal respiration	0.8	0.3	1.6	1.2	0.8	1.9	2.3	0.5	6.0	10.0	0.0006	A=G<F
DNA [mg/kg]	21.2	12.3	41.7	41.4	18.0	70.0	50.2	15.0	127.0	10.8	0.0004	A<G=F

[1)] One-way ANOVA for site characteristics, ANOVA with repeated measurement design including site as a random factor for yearly measured properties; [2)] Significant differences of pairwise tests between land-use types ($p < 0.05$): A - arable land, G - grassland, F - forest; [3)] MAT: mean annual temperature; [4)] MAP: mean annual precipitation; [5)] Soil properties and microbial parameters measured yearly during five years prior to the sampling of *Metarhizium* spp.
